# Impact of SARS-CoV-2 vaccines on Covid-19 incidence and mortality in the United States

**DOI:** 10.1371/journal.pone.0301830

**Published:** 2024-04-24

**Authors:** Fang Fang, John David Clemens, Zuo-Feng Zhang, Timothy F. Brewer

**Affiliations:** 1 Department of Epidemiology, Fielding School of Public Health, University of California at Los Angeles (UCLA), Los Angeles, CA, United States of America; 2 International Centre for Diarrhoeal Disease Research, Bangladesh, Dhaka, Bangladesh; 3 International Vaccination Institute (IVI), Seoul, the Republic of Korea; 4 Jonsson Comprehensive Cancer Center, UCLA, Los Angeles, CA, United States of America; 5 Department of Medicine, Center for Human Nutrition, UCLA David Geffen School of Medicine, University of California at Los Angeles (UCLA), Los Angeles, CA, United States of America; 6 Division of Infectious Diseases, UCLA David Geffen School of Medicine, Los Angeles, CA, United States of America; University of Ilorin, NIGERIA

## Abstract

**Background:**

Given the waning of vaccine effectiveness and the shifting of the most dominant strains in the U.S., it is imperative to understand the association between vaccination coverage and Severe Acute Respiratory Syndrome coronavirus 2 (SARS-CoV-2) disease and mortality at the community levels and whether that association might vary according to the dominant SARS-CoV-2 strains in the U.S.

**Methods:**

Generalized estimating equations were used to estimate associations between U.S. county-level cumulative vaccination rates and booster distribution and the daily change in county-wide Coronavirus 2019 disease (COVID-19) risks and mortality during Alpha, Delta and Omicron predominance. Models were adjusted for potential confounders at both county and state level. A 2-week lag and a 4-week lag were introduced to assess vaccination rate impact on incidence and mortality, respectively.

**Results:**

Among 3,073 counties in 48 states, the average county population complete vaccination rate of all age groups was 50.79% as of March 11^th^, 2022. Each percentage increase in vaccination rates was associated with reduction of 4% (relative risk (RR) 0.9607 (95% confidence interval (CI): 0.9553, 0.9661)) and 3% (RR 0.9694 (95% CI: 0.9653, 0.9736)) in county-wide COVID-19 cases and mortality, respectively, when Alpha was the dominant variant. The associations between county-level vaccine rates and COVID-19 incidence diminished during the Delta and Omicron predominance. However, each percent increase in people receiving a booster shot was associated with reduction of 6% (RR 0.9356 (95% CI: 0.9235, 0.9479)) and 4% (RR 0.9595 (95% CI: 0.9431, 0.9761)) in COVID-19 incidence and mortality in the community, respectively, during the Omicron predominance.

**Conclusions:**

Associations between complete vaccination rates and COVID-19 incidence and mortality appeared to vary with shifts in the dominant variant, perhaps due to variations in vaccine efficacy by variant or to waning vaccine immunity over time. Vaccine boosters were associated with notable protection against Omicron disease and mortality.

## Introduction

Since being recognized in December, 2019, the Severe Acute Respiratory Syndrome coronavirus 2 (SARS-CoV-2) pandemic has caused more than 479 million cases and six million deaths worldwide [1]. The United States has been particularly affected, with almost 80 million Coronavirus 2019 (COVID-19) infections and 972 thousand deaths reported as of March 25^th^, 2022 [2]. Though a number of non-pharmacologic prevention initiatives (NPIs) have been introduced to slow SARS-CoV-2 transmission [3], vaccines are now recognized as among the most effective means for preventing COVID-19 cases and deaths [4].

As of March 25, 2022, three vaccine preparations were authorized for use in the U.S. The BNT162b2 vaccine (Pfizer, Inc. and BioNTech) and the mRNA-1273 (Moderna) vaccine have full U.S. Food and Drug Administration (FDA) approval [5, 6], while JNJ-78436735 (Janssen Pharmaceuticals) is available under an emergency use authorization. All three vaccines are effective in preventing SARS-CoV-2 infections and COVID-19 associated diseases, hospitalizations, and deaths [7–9], though vaccine effectiveness wanes over time and breakthrough infections occur [4, 10].

Numerous studies have demonstrated that SARS-CoV-2 vaccines are effective in preventing COVID-19 infections and disease in individuals outside of clinical trials, including among health care workers, first responders, individuals attending ambulatory clinics, veterans, and in nursing homes [11–13]. However, vaccine waning has been reported, especially after 6 months being fully vaccinated [14–16]. Vaccine effectiveness also varies by SARS-CoV-2 variant. Omicron, the most recent predominant variant, evades infection- or vaccination-induced immunity more effectively than Delta variant, and correspondingly has higher rates of breakthrough infections or disease reported compared with other variants [14, 16–18]. With the previous recognition of waning vaccine immunity and the rise of Delta as the predominant SARS-CoV-2 variant in the U.S., the FDA authorized the emergency use of one or two boosters for COVID-19 vaccines, including the use of a heterologous booster [19–21]. Studies showed the effectiveness of vaccines improved after a booster dose, including against Omicron [16, 22, 23].

Despite the proven safety and effectiveness of these vaccines, a substantial minority of the adult U.S. population remains resistant to getting vaccinated [24]. To understand the impact of SARS-CoV-2 vaccines, it is imperative to evaluate the impact of vaccination on community-wide SARS-CoV-2 cases and COVID-19 disease, not just among those vaccinated—a concept popularly referred to as “herd immunity” [25]. Moreover, it is worth examining whether and by how much such an impact might differ corresponding to different dominant strains in the community. To investigate the impact of population percentages of SARS-CoV-2 vaccination on community-wide COVID-19 case and mortality rates, we undertook an ecological analysis of U.S. county vaccination rates on reported county COVID-19 cases and deaths, controlling for socioeconomic, demographic, comorbid conditions, rural/urban, air pollution, hospital capacity, and related factors, the introduction of NPIs and the presence of most prevalent strain in the U.S.

## Materials and methods

Poisson distribution with generalized linear models [26] were used to estimate associations between cumulative U.S. county-level vaccination rates of all age groups and the daily change in county-wide COVID-19 incidence and mortality between April 23^rd^, 2021, and March 25^th^, 2022. The dates represent when Delta was first recognized in the U.S. to the end of the study period. The analyses were divided into three periods to account for the most dominant strain in the U.S. during each period. The first period was from April 23^rd^ to July 2^nd^, 2021 before the Delta predominance and when Alpha was the most prevalent strain. Delta was responsible for the majority of reported U.S. COVID-19 cases from July 3^rd^ to December 1^st^, 2021. Between December 2^nd^, 2021 and March 25^th^, 2022, Omicron began circulating in the U.S. and replaced Delta to become the dominant strain.

All models were adjusted for the following potential confounders: annual average of ambient atmospheric particulate matter <2.5 μm in diameter (PM_2.5_) between 2000 and 2018, population density, poverty, education, proportions of White, proportions of male, proportion of population older than 65 years old, owner-occupied property, median house value, median household income, percentage of people without health insurance, proportion of people living in rural area, prevalence of tobacco smoking, and obesity. All covariates were measured at the county level. State-level variables for NPIs policies (facemask mandates, stay home orders) also were included in models. Table 1 summarizes data sources utilized in this study.

**Table 1:** Summary of data sources.

Sources	Description
Johns Hopkins University Center for Systems Science and Engineering Coronavirus Resource Center (CSSE) [2]	Cumulative county-level confirmed cases and deaths up to March 25^th^, 2022
Covid Act Now [27]	Cumulative county-level number of people completely vaccinated and number of completed vaccinated people receiving booster up to March 11^th^, 2022
Atmospheric Composition Analysis Group [28]	Annual average PM_2.5_ concentration between 2000 and 2018
The US Census/American Community Survey	County-level socioeconomic and demographic variables in 2020
The County Health Rankings & Roadmaps program [29]	Country-level behavioral variables and rural/urban status in 2020
Boston University of Public Health [30]	State-level policy of face masks mandates and stay home orders before July 2^nd^, 2022

County-level COVID-19 incidence and mortality data were obtained from the Johns Hopkins University, Center for Systems Science and Engineering Coronavirus Resource Center (CSSE). CSSE collects county-level confirmed numbers of cases and deaths of 3,342 counties across the U.S. from the U.S. Centers for Disease Control and Prevention (CDC) as well as state departments of health since January 21^st^, 2020 [2].

County-level vaccine data were obtained from Covid Act Now. These data are derived from the U.S. Department of Health and Human Services, the CDC, the New York Times, and official state and county dashboards. Data on vaccinations initiated, vaccination regimens completed and booster shots received were available for all 50 states [27]. To allow for the development of protective immunity after vaccination, a two-week lag was introduced after people were completely vaccinated (a person vaccinated on June 18^th^ was considered fully protected by July 2^nd^). The two-week lag also was used to account for time between exposure and development of COVID-19 disease. To assess the impact of vaccination on COVID-19 mortality, a four-week lag was used (vaccinated by September 2^nd^ to assess the impact on mortality on September 30^th^). In order to capture the immunity gained from the previous natural infection, county-level cumulative incidence was multiplied by the proportion of unvaccinated people among total infection during different stages of pandemic estimated by CDC [31]. A two-week and a four-week lags were applied to the cumulative incidence when assessing incidence and mortality outcomes, respectively. This was added to the fully vaccination rate and to the coverage of booster to estimate the holistic immunity in each county, by accounting for the immunity gained from both vaccination and from natural infection.

**Fig 1:**
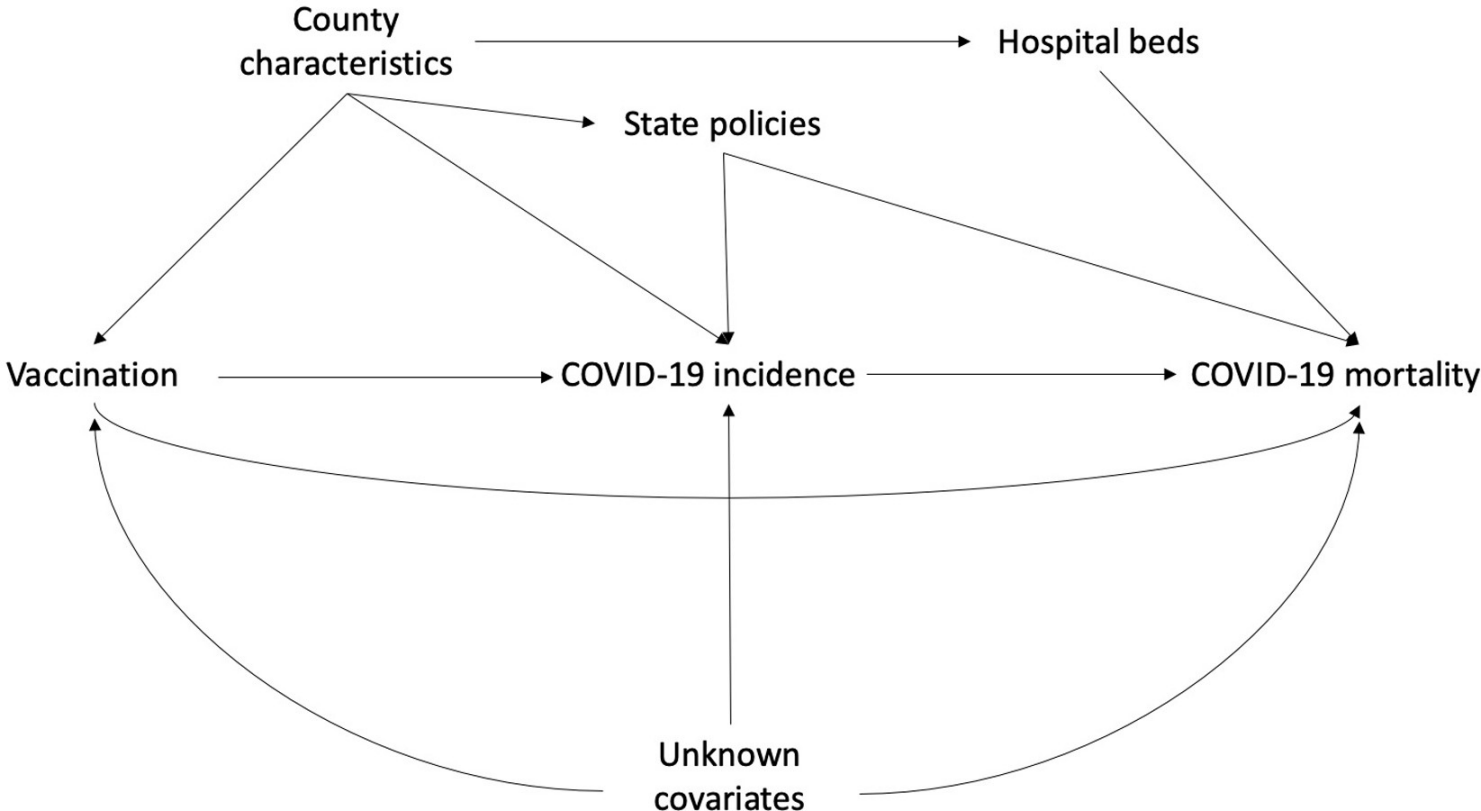
Directed acyclic graph. County characteristics include annual PM2.5 concentration between 2000 and 2018, percentage of adults smokers, percentage of obese adults, percentage of people living under poverty, population density, percentage of owner occupied properties, percentage of adults with less than high school education, percentage of White population, median household income, median house value, percentage of population over 65 years old, percentage of male, percentage of population without an insurance, and percentage of population living in rural area. State policies include stay home orders and facemask mandates.

Covariates were selected based on previous publications [3, 32] and availability of publicly accessible data. Fig 1 shows the directed acyclic graph applied in this study. County-level annual average of PM_2.5_ between the years 2000 and 2018, as well as county-level covariates, were available from the Atmospheric Composition Analysis Group [28]. County-level socioeconomic and demographic variables for 2020 were available from the US Census/American Community Survey. 2020 data on the prevalence of adult tobacco smoking and adult obesity and the proportion of people living in rural area were accessible through the County Health Rankings & Roadmaps program [29]. State averages were used to replace missing values for county-level prevalence for smoking and obesity. State-wide non-pharmacologic prevention policies, including facemask use and stay home orders, were obtained from the Boston University School of Public Health [30].

Counties with invalid Federal Information Processing Standards (n = 10), missing covariates (n = 98), missing vaccination status with a 2-week (n = 113) or a 4-week (n = 122) lag, and negative change in incidence (n = 48) or in mortality (n = 70) possibly due to data entry error were excluded. As a result, data from 3,073 counties in 48 states (excluding District of Columbia, Hawaii, and New Hampshire) were available to investigate the association between population vaccination rates and county-wide COVID-19 incidence and 3,042 counties in 48 states to investigate the association between vaccination rates and COVID-19 mortality. Among these eligible counties, 2,906 counties in 46 states and 2,876 counties in 46 states also reported percentage of people receiving a booster shot during the Omicron predominance and were utilized to assess the association between booster coverage and COVID-19 incidence and mortality, respectively. To assess the potential effect modification due to metropolitan status, stratified analyses by metropolitan status defined by Department of Agriculture [33] were performed. Relative risks (RR) and 95% confidence intervals (CI) are reported. Analyses were performed in SAS 9.4 (Cary, NC).

## Results

Among the 3,073 counties across 48 states, the average county total population complete vaccination rate was 50.79% as of March 11^th^, 2022. Counties with complete vaccination rates above the national median (49.8%) had higher median house values, higher median household incomes, higher population density, and less population living in rural area compared with counties with vaccination rates below 49.8%. These counties also were more likely to be located in states where a facemask policy or a stay-home order was ever issued before July 2^nd^, 2021 (Table 2).

**Table 2:** Characteristics of counties (n = 3073) in 48 states as of 3/25/2022.

	Mean (SD)		
	All	Vaccination rate ≤ 49.8% as of 3/11/2022	Vaccination rate > 49.8% as of 3/11/2022
No. of county in No. of states	3073 in 48 states	1542 in 40 states	1531 in 48 states
Incidence of COVID-19 as of 3/25/2022^a^, %	24.22 (5.70)	24.35 (5.66)	24.08 (5.73)
Incidence of COVID-19 as of 12/1/2021^a^, %	15.96 (3.98)	16.67 (3.80)	15.24 (4.03)
Incidence of COVID-19 as of 7/2/2021^a^, %	10.22 (3.07)	10.54 (2.93)	9.92 (3.16)
Mortality of COVID-19 as of 3/25/2022^b^, %	0.36 (0.16)	0.41 (0.16)	0.31 (0.15)
Mortality of COVID-19 as of 12/1/2021^b^, %	0.28 (0.14)	0.32 (0.14)	0.24 (0.13)
Mortality of COVID-19 as of 7/2/2021^b^, %	0.20 (0.11)	0.22 (0.11)	0.18 (0.11)
Full vaccination rate as of 3/11/2022^c^, %	50.79 (11.90)	42.48 (6.00)	60.17 (8.54)
Full vaccination rate as of 2/25/2022^c^, %	50.45 (11.83)	41.19 (5.96)	59.77 (8.48)
Full vaccination rate as of 11/17/2021^c^, %	44.51 (12.13)	35.92 (7.87)	53.17 (9.13)
Full vaccination rate as of 11/3/2021^c^, %	43.85 (12.32)	35.03 (8.17)	52.26 (9.33)
Full vaccination rate as of 6/18/2021^c^, %	32.06 (11.06)	24.82 (7.67)	38.99 (9.21)
Full vaccination rate as of 6/4/2021^c^, %	30.24 (10.64)	23.46 (7.62)	36.81 (8.89)
Booster rate as of 3/11/2022^c^, %	22.41 (8.24)	17.07 (4.16)	28.18 (7.65)
Booster rate as of 2/25/2022^c^, %	21.91 (8.12)	16.68 (4.12)	27.57 (7.57)
Average ambient PM_2.5_^d^, μg/m^3^	7.86 (2.18)	7.92 (2.16)	7.80 (2.19)
Ever smokers, %	17.48 (3.56)	18.32 (3.36)	16.63 (3.54)
Adult obesity, %	32.90 (5.42)	33.94 (5.01)	31.85 (5.61)
Population living in poverty, %	14.66 (6.18)	15.82 (5.90)	13.49 (6.24)
Population density, No. per square mile	206.44 (916.43)	59.56 (111.88)	354.38 (1276.72)
Owner occupied properties, %	72.08 (8.22)	73.18 (7.23)	70.97 (8.98)
Adults with less than high school education, %	12.74 (5.80)	14.33 (5.60)	11.13 (5.55)
White Americans population, %	77.05 (17.58)	79.63 (15.88)	74.45 (18.78)
African Americans population, %	8.83 (14.21)	8.56 (13.96)	11.04 (14.45)
Hispanic population, %	9.72 (13.63)	8.41 (11.59)	9.11 (15.31)
Median house value, ×$1,000	25.22 (66.44)	8.56 (10.31)	42.00 (90.52)
Median household income, ×$1,000	68.30 (16.73)	62.19 (11.42)	74.46 (18.84)
Population over 65 years old, %	19.28 (4.72)	19.54 (4.34)	19.02 (5.07)
Male, %	50.05 (2.38)	50.27 (2.65)	49.82 (2.05)
Uninsured population, %	9.48 (5.04)	10.81 (4.96)	8.14 (4.76)
Population living in rural, %	58.53 (31.38)	69.28 (26.18)	47.67 (32.46)
State stay-home order before 7/2/2021, n (%)			
Ever issued	2192 (71.33)	1013 (65.69)	1179 (77.01)
Never issued	881 (28.67)	529 (34.31)	352 (22.99)
State facemask policy before 7/2/2021, n (%)			
Ever issued	2299 (74.81)	991 (64.27)	1308 (85.43)
Never issued	774 (25.19)	551 (35.73)	223 (14.57)

^a^COVID-19 cases out of total population in each county

^b^COVID-19 deaths out of total population in each county

^c^Number of people fully vaccinated out of total population of all age groups in each county

^d^Annual average of PM_2.5_ between 2000 and 2018

When Alpha was the dominant strain in the U.S., each percentage increase in a county’s total population complete vaccination rate was associated with a 4% decrease in county-wide COVID-19 cases (relative risk (RR) 0.9607 (95% confidence interval (CI): 0.9553, 0.9661)) and with a 3% reduction in COVID-19 mortality (RR 0.9694 (95% CI: 0.9653, 0.9736)). However, county-level complete vaccine coverage was not associated with decreases in COVID-19 cases during the Delta (RR 0.9988 (95% CI: 0.9964, 1.0011)) and Omicron (RR 0.9969 (95% CI: 0.9919, 1.0019)) predominance. The association between complete vaccination rates and COVID-19 mortality declined to less than 0.1% (RR 0.9934 (95% CI: 0.9889, 0.9980)) when Delta accounted for the majority of reported cases in the U.S. between July 3^rd^ and December 1^st^, 2021. When Omicron began circulating, complete vaccination rate was associated with a slight increase of 0.6% in county-level COVID-19 mortality (RR 1.0061 (95% CI: 1.0022, 1.0101)). In contrast to the associations between complete vaccination rates and COVID-19 outcomes during the Omicron predominance, a 6% reduction in COVID-19 incidence (RR 0.9356 (95% CI: 0.9235, 0.9479)) and a 4% reduction in COVID-19 mortality (RR 0.9595 (95% CI: 0.9431, 0.9761)) were observed with each percentage increase in people receiving a booster shot at the county level. After accounting for the immunity gained from natural infection, the results remained similar (Table 3). Moreover, metropolitan status seems not to modify the association between vaccination and COVID-19 outcomes (Table 4).

**Table 3:** Adjusted relative risks of COVID-19 incidence and mortality associated with additional people fully vaccinated, with additional people receiving a booster dose, and with additional immunity per 100 population between April 23, 2021 and March 25, 2022 stratified by the most dominant variant.

	RR (95% Confidence Interval)		
Dominant variant	Alpha (4/23/2021–7/2/2021)	Delta (7/3/2021–12/1/2021)	Omicron (12/2/2021–3/25/2022)
Incidence^a^			
Fully vaccinated	0.9607	0.9988	0.9969
(n = 3,073 in 48 states)	(0.9553, 0.9661)	(0.9964, 1.0011)	(0.9919, 1.0019)
Booster	N/A	N/A	0.9356
(n = 2,906 in 46 states)			(0.9235, 0.9479)
Fully vaccinated and natural immunity	0.9738	1.0001	0.9863
(n = 3,073 in 48 states)	(0.9677, 0.9799)	(0.9978, 1.0025)	(0.9795, 0.9931)
Booster and natural immunity	N/A	N/A	0.9377
(n = 2,906 in 46 states)			(0.9268, 0.9488)
Mortality^b^			
Fully vaccinated	0.9694	0.9934	1.0061
(n = 3,042 in 48 states)	(0.9653, 0.9736)	(0.9889, 0.9980)	(1.0022, 1.0101)
Booster	N/A	N/A	0.9595
(n = 2,876 in 46 states)			(0.9431, 0.9761)
Fully vaccinated and natural immunity	0.9712	0.9981	1.0035
(n = 3,042 in 48 states)	(0.9656, 0.9767)	(0.9935, 1.0027)	(1.0002, 1.0069)
Booster and natural immunity	N/A	N/A	0.9660
(n = 2,876 in 46 states)			(0.9533, 0.9788)

^a^Model a adjusts for annual PM2.5 concentration between 2000 and 2018, percentage of adults smokers, percentage of obese adults, percentage of people living under poverty, population density, percentage of owner occupied properties, percentage of adults with less than high school education, percentage of White population, median household income, median house value, percentage of population over 65 years old, percentage of male, percentage of population without an insurance, percentage of population living in rural area, stay home orders before 7/2/2021 (ever/never) and facemask mandate before 7/2/2021 (ever/never); fully vaccination rate and booster rate were assessed two weeks prior the end of each period

^b^Model b adjusts for all covariates in model a; fully vaccination rate and booster rate were assessed four weeks prior the end of each period

**Table 4:** Adjusted relative risks of COVID-19 incidence and mortality associated with additional people fully vaccinated and with additional people receiving a booster dose per 100 population between April 23, 2021 and March 25, 2022 stratified by the most dominant variant and by metropolitan status.

	RR (95% Confidence Interval)		
Dominant variant	Alpha (4/23/2021–7/2/2021)	Delta (7/3/2021–12/1/2021)	Omicron (12/2/2021–3/25/2022)
Incidence^a^			
Metropolitan counties			
Fully vaccinated	0.9597	0.9991	0.9946
(n = 1,148 in 47 states)	(0.9536, 0.9658)	(0.9961, 1.0021)	(0.9881, 1.0010)
Booster	N/A	N/A	0.9324
(n = 1,063 in 45 states)			(0.9184, 0.9465)
Non-metropolitan counties			
Fully vaccinated	0.9623	0.9975	1.0049
(n = 1,924 in 45 states)	(0.9584, 0.9663)	(0.9957, 0.9993)	(1.031, 1.0067)
Booster	N/A	N/A	0.9606
(n = 1,842 in 43 states)			(0.9541, 0.9672)
Mortality^b^			
Metropolitan counties			
Fully vaccinated	0.9673	0.9947	1.0087
(n = 1,134 in 47 states)	(0.9627, 0.9720)	(0.9886, 1.0008)	(1.0044, 1.0130)
Booster	N/A	N/A	0.9569
			(0.9374, 0.9768)
(n = 1,049 in 45 states)			
Non-metropolitan counties			
Fully vaccinated	0.9781	0.9936	0.9973
(n = 1,907 in 45 states)	(0.9738, 0.9823)	(0.9903, 0.9969)	(0.9947, 0.9999)
Booster	N/A	N/A	0.9687
(n = 1,826 in 43 states)			(0.9609, 0.9765)

^a^Model a adjusts for annual PM_2.5_ concentration between 2000 and 2018, percentage of adults smokers, percentage of obese adults, percentage of people living under poverty, population density, percentage of owner occupied properties, percentage of adults with less than high school education, percentage of White population, median household income, median house value, percentage of population over 65 years old, percentage of male, percentage of population without an insurance, percentage of population living in rural area, stay home orders before 7/2/2021 (ever/never) and facemask mandate before 7/2/2021 (ever/never); fully vaccination rate and booster rate were assessed two weeks prior the end of each period

^b^Model b adjusts for all covariates in model a; fully vaccination rate and booster rate were assessed four weeks prior the end of each period

## Discussion

Data from 3,073 counties across 48 states demonstrates that the associations between county-level complete vaccination rate and COVID-19 incidence and mortality varied based on the most prevalent SARS-CoV-2 variant circulating in the U.S. between April 23^rd^, 2021 and March 25^th^, 2022 after adjusting for potential confounders. The protective associations between county-level complete vaccination rate and COVID-19 incidence and mortality were observed during the Alpha predominance, but such associations attenuated later when Delta or Omicron was the most prevalent strain in the U.S. However, after booster shots were available, the increase in the county percentage of people receiving a booster shot was associated with reduction in both COVID-19 incidence and mortality between December 2^nd^, 2021 and March 25^th^, 2022.

This study is among the first to show the population-wide association between SARS-CoV-2 vaccination rate and COVID-19 incidence and mortality stratified by the predominant strain circulating in the country. The results show that county-level vaccination rate has different associations with COVID-19 incidence and mortality during different periods in the U.S. The protection was highest shortly after COVID-19 vaccines became widely available while Alpha was the predominant circulating strain and declined in later periods. This pattern might be due to the waning effect of the vaccines against infection over time within the community. A meta-analysis showed that though vaccine effectiveness against SARS-CoV-2 infections was reduced, vaccine remained highly efficient in protecting people from severe diseases due to COVID-19 [15]. In addition, as vaccine uptake increased and cases declined, most states lifted their NPIs orders [30]. Without the protection of NPIs and given the waning of vaccine effectiveness, people became more susceptible to COVID-19 infection even when fully vaccinated. Besides the waning vaccine effectiveness, our results also suggest the association of vaccine coverage and COVID-19 incidence might depend on the most prevalent strain in the community. The protection of increased vaccination coverage against county-level COVID-19 incidence or mortality was not observed when Omicron circulation predominated, which has been shown to evade previous immunity more than Alpha or Delta [17]. However, the majority of COVID mortality occurred among unvaccinated people in the U.S. throughout our study period [34]. In the light of the waning vaccine effectiveness and breakthrough cases, a booster shot has been recommended. Individual-level and experimental data demonstrate that a third dose of mRNA COVID-19 vaccines increases vaccine efficacy [16, 17]. Similarly, our results also suggest that the increasing uptake of a booster shot is associated with the reduction in community COVID-19 cases and deaths. Moreover, this study considers the holistic immunity, not only gained from vaccination but also gained from the natural infection.

The study was subject to several limitations. First, ecologic study designs are vulnerable to the ecologic fallacy. Therefore, caution is required when interpreting the study results, especially when extrapolating population findings to the individual level. In addition, we cannot rule out the possibility of residual confounding even after controlling for numerous county-level and state-level covariates. Using COVID-19 reported cases may underestimate of the number of actual infections due to under-testing of asymptomatic patients, especially when self-tests became widely available. However, alternative estimates for cumulative incidence, such as seroprevalence [35], also have limitations including sampling bias, test sensitivity and specificity, and the progress of the pandemic [36]. Our analysis was not able to assess the impact of the three different vaccines currently available in the U.S. due to data availability, which likely had different efficacies. Although a detailed distribution of different variants in the U.S. was not available, we examined the associations stratified by the most dominant strain. Therefore, our results represent the associations between vaccine rates overall and COVID-19 incidence and mortality in the U.S. for vaccines as actually deployed and SARS-CoV-2 variants as they circulated during the period of our analysis.

## Conclusions

Nevertheless, this study is the first to estimate the association between complete vaccination rates and COVID-19 incidence and mortality in the U.S. general population using county-level data. This nation-wide study covers 3,073 counties in 48 states across the entire country, showing the population-based impact of increasing complete vaccination rates, as well as increasing percentage of those receiving a booster shot. Our results agree with the observation of waning effectiveness over time and higher infection breakthrough rates due to the Omicron variant. However, increasing the coverage of booster shot appear to be an effective way to protect individuals in the community and to potentially to achieve herd immunity.
